# Studying Regeneration Through History as a Way of Looking Forward

**DOI:** 10.1007/s10739-024-09769-5

**Published:** 2024-03-19

**Authors:** Kate MacCord, Jane Maienschein

**Affiliations:** The Marine Biological Laboratory, School of Life Sciences, Arizona State University, Tempe, Arizona 85287-4501 USA

## Motivation for this Essay

2024 is a good year for celebrating history of science. First comes the 100th year of the History of Science Society (HSS). Historian of Science George Sarton played a major role in establishing the Society, and he had clear views about what the field should include. He insisted that “the chief requisite for the making of a good chicken pie is chicken; nay, no amount of culinary legerdemain can make up for the lack of chicken. In the same way, the chief requisite for the history of science is intimate scientific knowledge; no amount of philosophic legerdemain can make up for its absence” (Sarton 1918, p. 194). From the beginning of the HSS, then, science played a central role. When one of us (Maienschein) served as HSS president in 2008–2009, she worked to include science and scientists in committees and activities, and the following president Paul Farber did as well.

2024 is also a new beginning for the *Journal of the History of Biology (JHB)*, with editors Betty Smocovitis and Nic Rasmussen at the helm since 2023. This journal, begun by Everett Mendelsohn and supported by Ernst Mayr, was always steeped in science. Mendelsohn explained that he started the journal and remained editor for 31 years to provide a place for historians of biology, with their interests in the ideas and practices of biological sciences, to publish (Mendelsohn 1968). Smocovitis and Rasmussen are carrying on the tradition of embedding the history with the science, so that the historical work the journal publishes is useful to both historians and biologists. Shortly before he died early in 2023, Mendelsohn told Maienschein that he was pleased with the continued emphasis on science along with history.

We may also point to the International Society for the History, Philosophy, and Social Studies of Biology (fondly known as Ishkabibble, or ISH for short) as a conjunction for history and science, along with its complementary fields. In 2024, *JHB* co-editor-in chief Smocovitis has become the president of ISH, MacCord is the program co-chair, and Maienschein is proud to have served as the first president for this organization and its embrace of science and the several fields that study it.

At this time when science is under attack politically, and history is often (falsely) considered irrelevant to current pressing issues, it is all the more important that we look at ways that history and science can work together. They can nudge each other to ask new questions, explore new areas, and thereby enhance and enrich both domains. History can be at once for science, with science, about science, and part of science, as we explain in the context of one particular example below. Looking at history and science simultaneously helps us look forward with perspective and reflection.

## Introduction

The idea that history of science has the potential to impact the sciences is not new. Historians have long made arguments about how our work can influence the sciences, including, but not limited to learning from past mistakes, providing clarity, provoking the imagination of scientists by providing a wider repertoire of ideas from which to choose, or shaping science education (Brush 1974; Chang 2011; Maienschein 2000; Maienschein et al. 2008; Matthews 2014). These arguments are often framed within disciplinary boundaries, such that the impact of history on science is akin to osmosis: historians produce history (sometimes in conference with scientists), and that history is then put out into the scholarly and/or public realms with the intention that scientists may take it up. In this sense, many (but not all) arguments about the impact of history on science are largely passive, describing how history (and historians) can be taken up by science (and scientists). The converse idea that science can impact history and shape historiography is far less widespread in the literature. The most prominent arguments on this side range from the complementary role of history for science that enables historians to rediscover ways of thinking that have been left out of modern scientific disciplines (Chang 2004, 2017), to the idea that re-creating experiments can reveal otherwise unknowable insights into the scientific process (Maienschein et al. 2008). So, what are we adding to these arguments?

In 2008, Maienschein and colleagues wrote that “[b]y working with biologists…historians of science can help identify and interpret the original assumptions and constraints underlying different models, theories, and practices” (Maienschein et al. 2008, p. 349, also see Maienschein 2001). Maienschein and colleagues made the point that historical methods can inform understanding of science in its relevant contexts, and such analysis carried out alongside scientists can shape what historical questions we ask and how we go about addressing those questions. We now want to build on this point to argue that historians of science can play a role in shaping the future of the sciences, and scientists can play a role in shaping the future of the history of science. To further this argument, we’ll dissect two claims, both of which cut both ways and impact both history and science. First, we claim that collaborations among historians of science and scientists provide a more robust understanding of the science, to the benefit of each. That science is the object of inquiry for historians of science is obvious by the very name of our field. That history is fundamental to all sciences is undeniably and demonstrably true (Creath 2010). Given these two points, collaborations among members of these two disciplines have a lot to offer each side in understanding the object of both of our interests, namely science.

Our second claim is that through such collaborations among historians and scientists we can uncover assumptions within science that yield both interesting historical and scientific questions that provide new and fruitful avenues of research. The unearthing of assumptions is a mainstay of the historian’s toolkit; we are trained to analyze, question, and prod our historical objects. Using somewhat different tools, so too are scientists trained to test assumptions. Our core argument and both of our points rely on active and immersive collaborations in which both the historian and the scientist are engaged in joint and iterative knowledge-making. What exactly this means, and how exactly this works, we will return to later in this essay as we provide evidence to support our claims. But, before we move to fleshing out our evidence, let’s begin with the hallmark of any good historical work: context. Our context is drawn from a collaborative project amongst historians, philosophers, and scientists based at the Marine Biological Laboratory in Woods Hole, Massachusetts, called the McDonnell Initiative.

## Context: The Origins of the McDonnell Initiative at the Marine Biological Laboratory

Marine laboratories have a long history, as well as a rich history of historians turning to their archives and working among the scientists in these institutions. One such place, the Marine Biological Laboratory in Woods Hole, Massachusetts (MBL), has been a convening place since 1888. Historical work has long been a feature of this institution—many historians have traveled to this scenic campus at the tip of Cape Cod to scour the archives and participate in courses, while many scientists have been inspired by the frenetic energy and scientific discoveries at the MBL to produce histories (see Lillie 1988; Zottoli and Seyfarth 2015). Around the centennial of the MBL, history became a fixture of the institution’s educational offerings (although historians had been working at the MBL long before) when Garland Allen and Jane Maienschein began the annual History of Biology seminars in 1987, as the first activity launching the MBL’s centennial year. Over the intervening years, hundreds of historians, philosophers, and scientists have attended this annual week-long seminar for intensive and immersive discussions about topics in the life sciences, from genetics to engineering life to regeneration.

In addition to the History of Biology seminar, the MBL houses a digital humanities initiative called the MBL History Project.^1^ This is a rich collection of digitized photographs and archival materials, online exhibits, video interviews with over 100 MBL scientists and community members, and records of those who have participated in the MBL’s world-famous courses every year. As part of the library of the MBL and its sibling, the Woods Hole Oceanographic Institution, this collection is easily available for anybody as an open access resource. The MBL History Project, which MacCord ran as the founding Project Manager, was designed to preserve and communicate the history of the science done at the MBL, providing reflections on what the MBL looked like in the past, to reflect upon into the future.

Preserving the history of the MBL through the MBL History Project led to questions about what the MBL could do, as an institution, to shape the future of the biology done there. One thing is obvious about the MBL: it is a wonderfully rich place for scientific discovery in different areas of biology, including regenerative biology and tissue engineering (the Eugene Bell Center for Regenerative Biology and Tissues Engineering), microbiomes and microbial communities (the Josephine Bay Paul Center for Comparative Molecular Biology and Evolution), and ecosystems ecology (the Ecosystems Center). While these three centers work together on various initiatives, especially focused on developing new research organisms and innovations in imaging, the centers do not come together in large, overarching research projects.

Susan Fitzpatrick, as president of the James S. McDonnell Foundation, challenged us to bring our approaches as historians to ask what kinds of research questions might draw together the MBL’s Centers and Initiatives. Fitzpatrick had herself first visited the MBL as a graduate student and had become enchanted, as we all are by the place and community around intense curiosity that makes discovery happen. Fitzpatrick asked, “is there anything in common across these different areas of research at the MBL? What might we learn by bringing them together? And how might history inform this process?” Thus began the McDonnell Initiative at the MBL.

In response to Fitzpatrick’s driving questions, we spent a great deal of time working with MBL scientists and thinking about what topics could span the MBL’s research centers and bring together regenerative biology, microbiomes/microbial communities, and ecosystems ecology. We arrived at one promising biological process: regeneration. The word regeneration invokes re-doing or re-building, perhaps regaining use of an injured limb, restoring the function of damaged nerves, or recovering one’s youthful energy. Whether through natural processes or the “miracles’’ of modern medicine, regeneration evokes images of replacing or repairing what was once there. Yet, regeneration can also refer to generating something again, where the processes and underlying materials remain but develop into something that is not just like the original. In this sense, regeneration provides a way for organisms to look forward and draw on creative processes. Furthermore, we suggest that this looking forward through regeneration can apply to all living systems. The McDonnell Initiative, then, is the effort of a collective of historians, philosophers, and scientists who have come together to understand regeneration—what it is, how it works, and how we understand it—across the scales of complex living systems. We are looking forward to what lessons we can draw that apply across systems and can help inform predictions about how to promote regeneration rather than the kind of failure that occurs when regeneration does not occur following a perturbed state.

Our regeneration project began in 2019, when MacCord, Maienschein, and Kathryn Maxson Jones organized a group to begin exploring questions surrounding what we mean by “regeneration” across all the different scales of life. We think we know what it means for organisms, but we came to realize that popular images of regenerating salamander tails can easily mislead us. Hydra or planarians simply do not behave the same way. There is something about their different individual systems, responding to their different contexts, that calls for more probing discussion of what we mean by those systems. Only when we begin to analyze each individual organism or its parts as a complex adaptive system at different levels did we begin to see bigger questions. So, what about ecosystems? Can they be seen as complex adaptive systems that also regenerate? At first, in 2019, our ecologists and historians of ecology thought not. Similarly, those studying microbial communities thought that those aggregations are just too different to represent the same kind of systems we have with individual organisms.

But the COVID pandemic has helped us to think differently and, we believe, to think better. Because we all had to stop rushing ahead doing more things actively through travel and working together in person, we started working more thoughtfully from different places. We developed a group of seven teams, each consisting of historians and philosophers of science (HPS scholars) and biologists, all asking what regeneration means in our respective areas of study. These teams crystallized around the study of:

1. Organisms: Jane Maienschein (HPS, Arizona State University/MBL) and Kate MacCord (HPS, Arizona State University/MBL).
2. Microbial communities and the impacts of rapid evolutionary change: S. Andrew Inkpen (HPS, Mount Allison University) and Ford Doolittle (Biology, Dalhousie University).
3. Ecosystems: Fritz Davis (HPS, Purdue University) and James P. Collins (Biology, Arizona State University).
4. Germlines: Kate MacCord (HPS, Arizona State University/MBL) and B. Duygu Özpolat (Biology, Washington University in St. Louis).
5. Neurons: Kathryn Maxson Jones (HPS, Purdue University) and Jennifer Morgan (Biology, MBL).
6. Stem cells and cancer: Lucie Laplane (HPS, CNRS/University of Paris 1/Gustave Roussy), Michel Vervoort^2^ (Biology, Institut Jacques Monod), and Eve Gazave (Biology, Institut Jacques Monod)
7. Complex adaptive systems generally including the planet earth in the context of the Anthropocene: Manfred Laubichler (HPS and Complexity Science, Arizona State University/Santa Fe Institute) and the whole group.

With monthly meetings, we share materials, try out ideas, work on written drafts, think about bigger issues, and always ask how study at one level might impact the others. This work has led to a series of small books for the University of Chicago Press, guided by editor Joseph Calamia, who has also become an intellectual contributor helping to think about ideas.

Three books of the series are so far published (Maienschein and MacCord 2022; Inkpen and Doolittle 2022; MacCord 2024), and the others are underway. Together, we keep thinking and working and learning from each other. In each case, we started with assumptions, discovered surprises, and moved toward new ideas. Coming together in person in 2019 allowed us to start thinking together, and over several intervening years we have been able to present, dissect, discuss, and refine ideas. Writing on our own, as historians tend to do, produces results. Biologists are less likely to do that kind of writing unless stimulated and working collaboratively with historians and philosophers. Workshopping ideas with other historians and biologists, then revising and rethinking produces what feel to us like better and more reflective results.

## Collaborations Among Historians and Scientists Cut Both Ways

Now that we’ve established the context, let’s return to our argument, namely that collaborations among historians and scientists cut both ways: *historians of science can play a role in shaping the future of the sciences, and scientists can play a role in shaping the future of the history of science*. Within this argument, we have laid out two main claims. First, that collaborations between historians and scientists provide a more robust understanding of the science, to the benefit of each. Second, that through such collaborations we can uncover assumptions within science that yield interesting historical questions that provide new and fruitful avenues of research.

In the introduction, we stated that both of our claims rely on active and immersive collaborations in which both the historian and the scientist are engaged in joint and iterative knowledge-making and promised to explicate what this means and how this works. Let’s start with what this means. By active and immersive collaborations, we mean that the participants work together on a shared problem or problems. Working together requires time, effort, embracing a shared language, and building up a relationship of trust. The point of such collaborations is to bring each area of expertise into the conversation, iteratively highlighting aspects of the problem that the collaborators understand in a different way and provoking each to consider what they think they know about the problem. This is the foundation of iterative knowledge-making. Now, how does this work? To answer this question, we’re best served by looking at the evidence that we have available to support our argument. This evidence is drawn from the working groups that make up the McDonnell Initiative at the MBL. We’ll briefly go through each working group whose book has been published, highlighting how the research of each has enriched understanding of the science, challenged assumptions, and provided new and intriguing areas for historical and scientific research.

### Organisms

Jane Maienschein and Kate MacCord. 2022. *What Is Regeneration?* Chicago: University of Chicago Press.

The first team, Maienschein and MacCord, started with existing literature about the history of regeneration research. Dead guys (biologists of the past) were our instructors, along with others at the MBL with whom we discussed ideas in the literature we read. Both popular presentations and the historical snippets that tend to appear at the beginning of scientific reviews pointed to salamander tails as a canonical example of regeneration. Cut off the tail, and “it” will grow back. The “it,” refers to the tail, as if it were the same tail. Maybe it’s a little smaller, maybe a slightly different color, but it’s presented as basically the same tail. In a parallel type of example, we have Prometheus, the mythological being, condemned to have an eagle pluck out his liver every day, and every night the liver – the same liver – would grow back. From such canonical examples, readers are easily led to believe that regeneration means regrowth of the same thing.

But that’s not really true. And our biological partners from the 18th century, René Antoine Ferchault de Réaumur and Abraham Trembley already thought so. Just look at their images of crayfish, earthworms, or hydra after they had cut off parts. Yes, something grows in the place where a part has been damaged or destroyed. Yes, if a hydra loses its head, it gets a new growth in the head location. But it might get two heads, or heads in different places, or something quite different like a distorted head. The living organism survives in the sense that it keeps living, and it has recovered in some ways, but it is not precisely the same. As one of our frequent scientific discussants, Alejandro Sánchez Alvarado at the Stowers Institute for Medical Research and the MBL, has repeatedly pointed out, Thomas Hunt Morgan knew this when he summarized existing knowledge about regeneration in his 1901 *Regeneration*. Morgan’s earthworms, planarians, and hydra also lose a head and then develop a head. But, again, he didn’t think of the head as the same thing. In fact, it might look rather different. As Morgan noted, it was not the part but rather the living system or the organism as a whole that was restored through the process of regeneration. Regeneration, then, can be thought of not simply as the restoration of a particular localized structure within an organism, but as a systemic response to injury.

As we look at current literature on regeneration, we see the tendency to think in terms of regeneration as a process that produces the same thing. Developmental biologists like to discover which genes are activated by injury and allow regeneration, and there is a tendency by many to assume that these are the genes active in the original developmental process as well. Similarly, recent work has focused on stem cells, both activating extant stem cells within a system and dedifferentiating various tissues into stem cells, to initiate regeneration. Here developmental biologists often ask what mechanisms stimulate the stem cells to produce the “right” processes and parts. Again, we witness that idea of “the” missing part being replaced.

Our historical work, and our collaborations with MBL scientists have thus led us to think of regeneration as a systems-level process, rather than a straightforward restoration of a part. This systems-focused vision of regeneration, in return, has forced all of us to think differently about what is necessary and sufficient for regeneration to occur within an organism, and even what may count as regeneration. We have found some biologists, notably Sánchez Alvarado, open to questions about what regeneration really means. We can now ask: in what sense does the process recover something that was lost in order to restore the system, but perhaps in different ways than what was there before? What might we learn by assuming that the regenerative process brings change and adaptation instead of return to sameness? Can we think of regeneration as a restorative process that looks forward to the continuity of the complex system? Seeing organisms in terms of complex adaptive systems, subject to many forces and factors both internal and environmental, may bring advances in the biology that may lead to the kinds of medical applications regeneration biologists long to find. And such questions inform our history by shifting our focus from replacement of parts to restoration of integrity.

### Microbial Communities

AndrewS. Inkpen and W. Ford Doolittle. 2022. *Can Microbial Communities Regenerate? Uniting Ecology and Evolutionary Biology*. Chicago:University of Chicago Press.

Inkpen and Doolittle have brought multiple perspectives to their exploration of microbial communities. Their first reaction to the idea that microbial communities might regenerate was “no, no, it doesn’t happen.” Because a community of microbes changes constantly, as some species of microbes replace others, they had explained in earlier work that what matters is “not the singer, but the song.” (Doolittle and Booth 2017; Doolittle and Inkpen 2018). The individual species and microbes can change, but the community with its “singers” remains. What matters is the function rather than the structure in this case. Because microbial community structures are subject to so much change, Inkpen and Doolittle couldn’t draw a direct parallel between regenerating a microbial community and canonical examples of regeneration like salamander tails. Thus, their baseline assumption for microbial communities was that those communities are not subject to regeneration.

On reflection, and after a great deal of discussions with members of the McDonnell Initiative, they began to consider in what ways microbial communities actually can regenerate. They came to the idea that maybe it is really possible to see microbial communities as regenerating, as long as we look at the phenomenon in light of evolution and focus on the collective functions and not the community structure. Their volume *Can Microbial Communities Regenerate?* took on the theme of “uniting ecology and evolutionary theory.” Understanding communities in terms of complex systems adapted to the environment and having evolved over time leads to a more nuanced understanding of both the systems and what might be meant by *health*. So, yes, there are senses in which these communities regenerate, and thinking about microbial communities in terms of regeneration has led them to consider how evolutionary processes can work to sustain community functions rather than structures. This thinking requires a shift in both the science and its interpretation, away from focusing the emphasis on particular individual species of microorganisms and their impacts to looking at systems. The science is informed by explicit recognition that focusing on the whole system gives different answers than focusing only on individual components; and understanding among HPS scholars also shifted when they expanded the domain of literature they considered relevant to interpretations of microbial communities as complex regenerating systems away from looking at particular details to looking at the whole microbial community system and its interactions over time.

### Germline

Kate MacCord. 2024. *How Does Germline Regenerate?* Chicago: Chicago University Press.

Germline (i.e., the germ cell lineage that contributes to reproduction) is not an obvious place to explore our understanding of regeneration because it is commonly held within biology that the germline does not regenerate except under very limited circumstances. These limited circumstances occur when germ cells are present and able to regenerate whenever germ cells have been damaged or removed. This thinking about germline regeneration stems from the understanding that germ cells (reproductive cells and their direct lineage of antecedents) and somatic cells (every cell in the body that is not a germ cell) are separate and distinct. And yet, by the time the McDonnell Initiative had started, biologist B.D. Özpolat and others had shown that germline does regenerate in some organisms, seemingly without the involvement of germ cells.

MacCord and Özpolat thus started their collaboration by examining why the scientific community held this belief about germline. This exploration led to questions about how we understand germline and germ cells, both now and in the past, and how we know what we know. As MacCord sifted through the historical literature and discussed it with Özpolat, it became clear that the modern conception of germline and germ cells, as something separate and distinct from the rest of the body, was based on a number of assumptions, some of which are historically entrenched (MacCord 2024).

One such assumption is that organisms are constrained by what is called the Weismann barrier. The idea of the Weismann barrier holds that there is a simple and strict relationship between germ cells and somatic cells, such that germ cells can give rise to somatic cells, but somatic cells can never become germ cells. The Weismann barrier concept is not new—it was developed by cytologist Edmund Beecher Wilson in his 1896 text *The Cell in Development and Inheritance* based on a fundamental mischaracterization of the theoretical work of August Weismann and his germ plasm theory (Wilson 1896; Churchill 2015; Griesemer and Wimsatt 1989; MacCord 2024). Over more than a century of biological research, the Weismann barrier became a core tenet of organismal biology, in part thanks to its uptake by practitioners within the nascent field of genetics, and developmental biologists married to theory above evidence (MacCord 2024). MacCord realized that germline regeneration was the perfect place to test the limits of the Weismann barrier. Afterall, when germline regenerates, those cells have to come from somewhere.

Today, Özpolat’s research program, and that of several others, has indicated that germline actually can regenerate from somatic cells. Working together, MacCord and Özpolat realized the far-reaching implications of such a simple shift in thinking about the relationship between these two kinds of cells. All of our genome editing policies are guided by the notion that the Weismann Barrier is inviolable—that somatic cells can never become germ cells. This is why we have separate policies for heritable (germline) and non-heritable (somatic) genome editing. But if somatic cells can become germ cells, and somatic cell genome editing is used on humans (which it is), our separate genome editing policies built on the inviolability of the Weismann Barrier have introduced the potential for inadvertent heritable genome editing. Thinking about germline regeneration—how it works, where the cells come from, under what conditions it happens—thus can greatly impact both a core tenet of biology and the foundations of human genome editing. Only by working with Özpolat, whose lab was already beginning to test the boundaries of an inviolable Weismann barrier, was MacCord able to see the historical work needed.

## Conclusions

To conclude, let’s return to our argument: historians of science can play a role in shaping the future of the sciences, and scientists can play a role in shaping the future of the history of science. As we can see through the McDonnell Initiative working groups, collaborations can indeed enrich our understanding of science, uncover and challenge assumptions within modern and past biology, and open new and intriguing areas of research for the historian and scientist alike. These collaborations can cut both ways. Through active and iterative knowledge-making, historians and scientists both benefit from the knowledge and skill sets of each. Therefore, these kinds of collaborations have immense value to our field on their own, but can also put the history of science in the position of shaping the direction of science.

## Footnotes

1 See http://history.archives.mbl.edu.

2 Our friend and collaborator, Michel Vervoort, passed away on December 8, 2022.
